# GENDER DIFFERENCES IN THE IMPACT OF COVID-19’S HARDSHIP ON DEPRESSIVE SYMPTOMATOLOGY AMONG MEXICAN OLDER ADULTS

**DOI:** 10.1093/geroni/igae098.2675

**Published:** 2024-12-31

**Authors:** Sirena Gutierrez, Rebeca Wong, Sadaf Milani

**Affiliations:** University of California, San Francisco, San Francisco, California, United States; The University of Texas Health Sciences Center in San Antonio, San Antonio, Texas, United States; The University of Texas Medical Branch, Galveston, Texas, United States

## Abstract

Older women in Mexico report depressive symptoms more often than men. Amidst the COVID-19 pandemic, the exacerbation of mental health inequities among older adults remains unclear. We investigated whether the association between experiencing hardship and depressive symptoms varied by attributing these events to the pandemic. We used data from the Mexican Health and Aging Study (2018/2021) and included participants aged 50+ (n=13,046). Participants self-reported whether they experienced the following hardship events: child or spousal death, caregiving for a sick family member, worsened finances, or job loss. If they said yes, they could attribute the event to COVID-19, resulting in two binary measures of hardship events (0 vs. 1+), pandemic-related or non-pandemic. Our primary outcome was a 9-item depressive symptom score. We used generalized estimating equations models adjusting for sociodemographic covariates and a time fixed effect. Gender heterogeneity was examined with stratified models and multiplicative interaction terms. Participants averaged 63.7 years, and 56.3% were women. Both non-pandemic (b=0.38, 95% confidence interval [CI]: 0.30, 0.46) and pandemic-related (b=0.43, 95% CI: 0.31, 0.56) hardship were associated with greater depressive symptoms. The magnitude of the associations was stronger for women (b=0.44, 95% CI: 0.34, 0.55 vs. b=0.29, 95% CI: 0.18, 0.40 for non-pandemic hardship; b=0.49, 95% CI: 0.32, 0.66 vs. b=0.34, 95% CI: 0.15, 0.53 for pandemic-related hardship). However, the gender difference was only statistically significant for non-pandemic hardship (interaction p=0.042). Understanding mechanisms for gender differences in the impact of the pandemic on the mental health of older adults is essential for targeted interventions.

